# Publisher Correction to: Global Health Research and Policy, volume 4

**DOI:** 10.1186/s41256-019-0114-2

**Published:** 2019-08-09

**Authors:** Global Health Research and Policy

**Affiliations:** London, UK

**Publisher correction to: Global Health Research and Policy, volume 4**

An error occurred during the publication of a number of articles *Global Health Research and Policy*. Several articles were published in volume 4 with a duplicate citation number.

In this correction article the old and new citation metadata are published in Table 1.

**Table 1:** Overview of incorrect and correct citation metadata

DOI	Incorrect citation number	Correct citation number
10.1186/s41256-019-0106-2	1	16
10.1186/s41256-019-0107-1	2	18
10.1186/s41256-019-0108-0	3	17
10.1186/s41256-019-0109-z	4	15

The original articles have been updated. The publisher apologizes for the inconvenience caused to our authors and readers.

